# Supplementary material for: Imitation dynamics on networks with incomplete information
Source: Nat Commun. 2023 Nov 17;14:7453. doi: 10.1038/s41467-023-43048-x (PMC10656501; doi:10.1038/s41467-023-43048-x)
Supplement: Supplementary file 1 — Supplementary Information [file 41467_2023_43048_MOESM1_ESM.pdf]

Supplementary Information for

Imitation dynamics on networks with incomplete information

Xiaochen Wang, Lei Zhou, Alex McAvoy, & Aming Li

Contents

1 Overview **2**

2 Model **2**

2.1 Network structure 2

2.2 Discrete-time absorbing Markov chain 2

2.3 Games and payoffs 2

2.4 Imitation with incomplete social information (IMisi) 3

3 Conditions for the success of cooperators **4**

3.1 General condition under weak selection 4

3.2 Condition under IMisi 4

3.3 Specific conditions for different game interactions 6

4 The threshold of the network clustering coefficient **7**

5 Supplementary figures **9**

Supplementary Note 1: Overview

This supplementary text is organised as follows. In Section 2, we introduce the game model and evolutionary dynamics on regular networks. In Section 3, we derive the conditions under which cooperation is favoured by natural selection and provide a detailed analysis of different game types. In Section 4, we provide a method to construct a series of regular networks with increasing clustering coefficients, and present the existence of the threshold of the clustering coefficient where the impact of incomplete information is reversed. In Section 5, supplementary figures are presented.

Supplementary Note 2: Model

2.1 Network structure

We consider a population of fixed size N. The interaction and replacement relations between individuals are depicted by an undirected regular network G. On the network, each individual i has d neighbours. Let W be the adjacency matrix of network G. If node i and j are connected, the weight between them is $w_{ij} = w_{ji} = 1$; otherwise, $w_{ij} = w_{ji} = 0$. The total weight of individual i is $w_i = \sum_{j \in G} w_{ij} = d$.

2.2 Discrete-time absorbing Markov chain

In the game, individuals can use one of the two strategies, cooperation (C) or defection (D). The state of the system can be depicted as a binary vector $\mathbf{x} = (x_1, x_2, \cdots, x_N)^{\mathrm{T}} \in \{0,1\}^N$, where $x_i = 1$ indicates that individual i is a cooperator and $x_i = 0$ a defector. The evolutionary process we consider here can be modelled by a discrete-time Markov chain S_n where the size of the state space is $\mathcal{M} = 2^N$. Specifically, this Markov chain will eventually end in two absorbing states where all individuals use the same strategy, namely, the all-C ($x_i = 1$ for all $i \in G$) or the all-D state ($x_i = 0$ for all $i \in G$). Based on this, we calculate the fixation probability of cooperators, ρ_{C}, namely, the probability that a uniformly randomly placed cooperator reaches the all-C state in a population full of defectors. Similarly, we also calculate ρ_{D} which denotes the probability that a uniformly randomly placed defector reaches the all-D state in a population full of cooperators. To tell whether natural selection favours cooperation over defection, we compare ρ_{C} with ρ_{D}: if $\rho_{\mathrm{C}} > \rho_{\mathrm{D}}$, cooperation is favoured over defection; if $\rho_{\mathrm{C}} < \rho_{\mathrm{D}}$, defection is favoured over cooperation.

2.3 Games and payoffs

At each time step, each individual i plays games by either cooperating (C) or defecting (D). Two types of game interactions are considered: donation game and public goods game. Under the donation game, a cooperator pays a cost c to offer its opponent a benefit b, and a defector pays nothing and provides no benefit. Such pairwise interaction can be depicted by the following payoff matrix

$$\begin{array}{cc} & \begin{array}{cc} \mathrm{C} & \mathrm{D} \end{array} \\ \begin{array}{c} \mathrm{C} \\ \mathrm{D} \end{array} & \begin{pmatrix} b-c & -c \\ b & 0 \end{pmatrix} \end{array}. \tag{S1}$$

Let us consider the random walks on G in discrete time. For a random walk on the regular network G, the probability of a one-step walk from node i to node j is $p_{ij} = w_{ij}/d$. We denote the probability that an m-step random walk goes from node i to j as $p_{ij}^{(m)}$. Here, $p_{ij}^{(m)}$ is the (i,j)-th entry of the matrix W^m/d^m. There is a unique stationary distribution $\lim_{m\to\infty} p_{ij}^{(m)} = 1/N$ for random walks on regular networks. Let $f_i^{(m)}$ be the

expected average payoff of an individual at the end of an m-step random walk from individual i on the regular network G. Under donation games, this average payoff is

$$f_i^{(m)} = -cx_i^{(m)} + bx_i^{(m+1)}, \tag{S2}$$

where $x_i^{(m)} = \sum_{j\in G} p_{ij}^{(m)} x_j$ represents the probability that an individual at the end of an m-step random walk from individual i is a cooperator.

In a public goods game, cooperators donate c to the common pool that generates a benefit rc, where r is the multiplication factor. And defectors contribute nothing. All the benefits are accumulated and then distributed evenly to all the participants. In an n-player public goods game ($1 < r < n$), when n_{C} ($0 \leq n_{\mathrm{C}} \leq n$) participants cooperate, the average payoffs that a defector and a cooperator receive are

$$f_{\mathrm{D}}(n_{\mathrm{C}}) = \frac{rn_{\mathrm{C}}}{n}c,$$

$$f_{\mathrm{C}}(n_{\mathrm{C}}) = f_{\mathrm{D}}(n_{\mathrm{C}}) - c,$$

respectively. Without loss of generality, we set $c = 1$. On a regular network with degree d, the expected average payoff of an individual at the end of an m-step random walk from individual i is

$$\begin{aligned} f_i^{(m)} &= r\left(\frac{d}{d+1}x_i^{(m+1)} + \frac{1}{d+1}x_i^{(m)}\right) - x_i^{(m)} \\ &+ rd\left(\frac{d}{d+1}x_i^{(m+2)} + \frac{1}{d+1}x_i^{(m+1)}\right) - dx_i^{(m)} \\ &= r\frac{d^2}{d+1}x_i^{(m+2)} + 2r\frac{d}{d+1}x_i^{(m+1)} + \left[r\frac{1}{d+1} - (d+1)\right]x_i^{(m)}. \end{aligned} \tag{S3}$$

The first row represents the payoff obtained in the game organised by individual i and the second row is the payoff obtained in the games organised by its neighbours. For example, the first term of the first row $r\left(\frac{d}{d+1}x_i^{(m+1)} + \frac{1}{d+1}x_i^{(m)}\right)$ corresponds to the expected benefits obtained from the cooperative neighbours and the focal individual itself. And the second term $x_i^{(m)}$ is the expected cost for the focal individual to cooperate. After the game interactions, the payoffs f_i are transformed into fitness F_i by the mapping $F_i = \exp(\delta f_i)$, where $\delta \geq 0$ is the selection intensity.

2.4 Imitation with incomplete social information (IMisi)

After the game interactions, a random individual is chosen to update its strategy. Here, we introduce an update rule called "imitation with incomplete social information" (IMisi) to characterize the imitation-based update process under incomplete information. Under IMisi updating, the probability that an individual i imitates the strategy of individual j is

$$p_{i\to j} = \begin{cases} \dfrac{(1-\theta)/sF_j}{(1-\theta)/s\sum_{l\in\Omega_i^{(s)}} F_l + \theta F_i}, & j \in \Omega_i^{(s)}, \\ \dfrac{\theta F_j}{(1-\theta)/s\sum_{l\in\Omega_i^{(s)}} F_l + \theta F_i}, & j = i, \end{cases} \tag{S4}$$

where s is the number of neighbours consulted, $\Omega_i^{(s)}$ denotes the set of s neighbours that individual i randomly selects, and $\theta \in [0, 1)$ is called the personal information weight and represents the relative importance of personal information to social information for strategy updating.

Supplementary Note 3: Conditions for the success of cooperators

3.1 General condition under weak selection

Let $D(\mathbf{x})$ be the expected instantaneous rate of change in the frequency of strategy C. $D(\mathbf{x}) = \sum_{i\in G} x_i(b_i(\mathbf{x}) - d_i(\mathbf{x}))$, where $b_i(\mathbf{x})$ is the probability that i replaces one of its neighbours and $d_i(\mathbf{x})$ is the probability it is replaced by its neighbours [1]. Under weak selection $0 < \delta \ll 1$, the fixation probabilities of cooperators and defectors are given by $\rho_{\mathrm{C}} = \frac{1}{N} + \delta \left\langle \frac{\partial}{\partial\delta} D \right\rangle^{\circ} + \mathcal{O}(\delta^2)$, and $\rho_{\mathrm{D}} = \frac{1}{N} - \delta \left\langle \frac{\partial}{\partial\delta} D \right\rangle^{\circ} + \mathcal{O}(\delta^2)$, respectively [1]. Here, $\langle\cdot\rangle^{\circ}$ means to take the average over the neutral drift ($\delta = 0$), i.e., for any function of the state $g(\mathbf{x})$ and any initial state $\mathbf{x}_0$, $\langle g\rangle^{\circ} = \sum_{t=0}^{\infty} \mathbb{E}^{\circ}_{\mathbf{x}_0}[g(\mathbf{x}(t))]$. Note that $\langle D(\mathbf{x})\rangle^{\circ} = 0$ for all $\mathbf{x} \in \{0,1\}^N$ [1]. The condition for cooperation to be favoured over defection is

$$\left\langle \frac{\partial}{\partial\delta} D(\mathbf{x}) \right\rangle^{\circ} = \left\langle \frac{\partial}{\partial\delta} \sum_{i\in G} x_i \left(b_i(\mathbf{x}) - d_i(\mathbf{x})\right) \right\rangle^{\circ} > 0. \tag{S5}$$

3.2 Condition under IMisi

We introduce two sets of selected neighbours of an individual j: $\mathcal{N}_j$ is the set of all possible $\Omega_j^{(s)}$ under a given s and the size of $\mathcal{N}_j$ is $\binom{d}{s}$; $\mathcal{N}_j^i$ is the set of $\Omega_j^{(s)}$ which contains individual i. Obviously, $\mathcal{N}_j^i \subseteq \mathcal{N}_j$. Here, we use $\langle\cdot\rangle_{\mathcal{N}}$ to represent the average taken over the set $\mathcal{N}$. According to Eq. (S4),

$$b_i(\mathbf{x}) = \frac{1}{N} \sum_{j\in\Omega_i^{(d)}} \left\langle \frac{s}{d} \frac{(1-\theta)/sF_i}{(1-\theta)/s\sum_{l\in\Omega_j^{(s)}} F_l + \theta F_j} \right\rangle_{\mathcal{N}_j^i}, \tag{S6}$$

where $\Omega_i^{(d)}$ is the set of the neighbours of individual i. Eq. (S6) gives the expected probability that individual i replaces its neighbours. To calculate this probability, one of individual i's neighbours, individual j, needs to be selected to change its strategy (with probability $1/N$). With probability s/d, individual i's information is known by individual j. Then, individual i replaces individual j with probability $[(1-\theta)/s]F_i/[(1-\theta)/s\sum_{l\in\Omega_j^{(s)}} F_l + \theta F_j]$. Averaging over all possible neighbours of individual i, we obtain Eq. (S6). Similarly, we have the expected probability that individual i is replaced by its neighbours, which reads

$$d_i(\mathbf{x}) = \frac{1}{N} \left\langle \frac{(1-\theta)/s\sum_{j\in\Omega_i^{(s)}} F_j}{(1-\theta)/s\sum_{j\in\Omega_i^{(s)}} F_j + \theta F_i} \right\rangle_{\mathcal{N}_i}. \tag{S7}$$

Substituting Eqs. (S6-S7) into condition (S5), we obtain that under weak selection, the condition for coop-

eration to prevail over defection under the IMisi rule is

$$\left\langle \frac{\partial}{\partial \delta} D \right\rangle^{\circ} = \left\langle \sum_{i \in G} x_i \cdot \frac{\partial}{\partial \delta} \left(\frac{1}{N} \sum_{j \in \Omega_i^{(d)}} \left\langle \frac{s}{d} \frac{(1-\theta)/s F_i}{(1-\theta)/s \sum_{l \in \Omega_j^{(s)}} F_l + \theta F_j} \right\rangle_{\mathcal{N}_j^i} - \frac{1}{N} \left\langle \frac{(1-\theta)/s \sum_{j \in \Omega_i^{(s)}} F_j}{(1-\theta)/s \sum_{j \in \Omega_i^{(s)}} F_j + \theta F_i} \right\rangle_{\mathcal{N}_i} \right) \right\rangle^{\circ}$$

$$= \left\langle \sum_{i \in G} \frac{1}{N} x_i \left(\frac{s}{d} \sum_{j \in \Omega_i^{(d)}} \left\langle \frac{(1-\theta)/s f_i [(1-\theta)/s \sum_{l \in \Omega_j^{(s)}} F_l + \theta F_j]}{[(1-\theta)/s \sum_{l \in \Omega_j^{(s)}} F_l + \theta F_j]^2} - \frac{(1-\theta)/s F_i [(1-\theta)/s \sum_{l \in \Omega_j^{(s)}} f_l + \theta f_j]}{[(1-\theta)/s \sum_{l \in \Omega_j^{(s)}} F_l + \theta F_j]^2} \right\rangle_{\mathcal{N}_j^i} \right.\right.$$

$$\left.\left. - \left\langle \frac{(1-\theta)/s \sum_{j \in \Omega_i^{(s)}} f_j [(1-\theta)/s \sum_{j \in \Omega_i^{(s)}} F_j + \theta F_i]}{[(1-\theta)/s \sum_{j \in \Omega_i^{(s)}} F_j + \theta F_i]^2} - \frac{(1-\theta)/s \sum_{j \in \Omega_i^{(s)}} F_j [(1-\theta)/s \sum_{j \in \Omega_i^{(s)}} f_j + \theta f_i]}{[(1-\theta)/s \sum_{j \in \Omega_i^{(s)}} F_j + \theta F_i]^2} \right\rangle_{\mathcal{N}_i} \right) \right\rangle^{\circ}$$

$$= \left\langle \sum_{i \in G} \frac{x_i}{N} \left\{ (1-\theta)[(1-\theta) + 2\theta] f_i - \frac{1}{d} \theta (1-\theta) \sum_{j \in \Omega_i^{(d)}} f_j - \frac{1}{s} (1-\theta) \theta \left\langle \sum_{j \in \Omega_i^{(s)}} f_j \right\rangle_{\mathcal{N}_i} \right.\right.$$

$$\left.\left. - \frac{1}{sd} (1-\theta)^2 \sum_{j \in \Omega_i^{(d)}} \left\langle \sum_{l \in \Omega_j^{(s)}} f_l \right\rangle_{\mathcal{N}_j^i} \right\} \right\rangle^{\circ} > 0. \tag{S8}$$

Here, $\left\langle \sum_{j \in \Omega_i^{(s)}} f_j \right\rangle_{\mathcal{N}_i} / s = \sum_{j \in \Omega_i^{(d)}} f_j / d = f_i^{(1)}$ denotes the expected payoff of the neighbours of i since the s neighbours are randomly selected from all the d neighbours. The last term $\sum_{j \in \Omega_i^{(d)}} \left\langle \sum_{l \in \Omega_j^{(s)}} f_l \right\rangle_{\mathcal{N}_j^i} / (sd)$ denotes the expected payoff of an individual that is at the end of a special two-step random walk from the node occupied by individual i. Note that in the second step, the random walk can only go to s selected neighbours, and individual i must be selected. Under IMisi updating, this expected payoff is

$$\frac{1}{sd} \sum_{j \in \Omega_i^{(d)}} \left\langle \sum_{l \in \Omega_j^{(s)}} f_l \right\rangle_{\mathcal{N}_j^i} = \frac{1}{sd} \sum_{j \in \Omega_i^{(d)}} \left\langle \sum_{l \in \Omega_j^{(s)}, l \neq i} f_l + f_i \right\rangle_{\mathcal{N}_j^i} = \frac{1}{sd} \sum_{j \in \Omega_i^{(d)}} \left(\left\langle \sum_{l \in \Omega_j^{(s)}, l \neq i} f_l \right\rangle_{\mathcal{N}_j^i} + f_i \right)$$

$$= \frac{1}{s} \left[(s-1) \left(\frac{d f_i^{(2)} - f_i^{(0)}}{d-1} \right) + f_i^{(0)} \right] = \frac{s-1}{s} \left(\frac{d f_i^{(2)} - f_i^{(0)}}{d-1} \right) + \frac{f_i^{(0)}}{s}. \tag{S9}$$

Note that Eq. (S9) is different from the average payoff of s neighbours that are known beforehand and fixed during the evolution. This is because when s neighbours are randomly selected if individual i competes, its information must have been known by its neighbour who is updating its strategy. Hence, the expected payoff in Eq. (S9) must include the payoff of individual i.

Employing Eq. (S9), we further simplify condition (S8) and get

$$\left\langle \frac{\partial}{\partial \delta} D \right\rangle^{\circ} = \left\langle \sum_{i \in G} \frac{(1-\theta) x_i}{N} \left\{ [(1-\theta) + 2\theta] f_i^{(0)} - 2\theta f_i^{(1)} - (1-\theta) \left(\frac{s-1}{s} \left(\frac{d f_i^{(2)} - f_i^{(0)}}{d-1} \right) + \frac{f_i^{(0)}}{s} \right) \right\} \right\rangle^{\circ}$$

$$= \left\langle \sum_{i \in G} \frac{(1-\theta) x_i}{N} \left[2\theta (f_i^{(0)} - f_i^{(1)}) + (1-\theta) \frac{(s-1)d}{(d-1)s} (f_i^{(0)} - f_i^{(2)}) \right] \right\rangle^{\circ} > 0 \tag{S10}$$

$$\iff \left\langle \sum_{i \in G} \frac{x_i}{N} \left[2\theta \frac{s}{d} (f_i^{(0)} - f_i^{(1)}) + (1-\theta) \frac{s-1}{d-1} (f_i^{(0)} - f_i^{(2)}) \right] \right\rangle^{\circ} > 0. \tag{S11}$$

We now have the condition (8) in the main text, with the notation $f_i^{(n)}$ replaced by $\overline{u}^{(n)}$.

3.3 Specific conditions for different game interactions

To calculate condition (S11), we introduce the coalescing random walk, which is a collection of random walks that progress independently until two walks meet [1]. Backwards in time, this is a process of tracing ancestors. Let τ_{ij} denote the expected coalescence time between i and j under the discrete-time coalescing random walk. Suppose i and j are the two ends of a random walk of length m. Analogous to other imitation-based update rules, we have $\tau_{ii} = 0$ and

$$\tau_{ij} = \frac{1}{1-\theta} + \frac{1}{2}\sum_{k=1}^{N} p_{ik}^{(1)} \tau_{kj} + \frac{1}{2}\sum_{k=1}^{N} p_{jk}^{(1)} \tau_{ik}, \tag{S12}$$

for $i \neq j$. We denote $\tau^{(m)} = \sum_{i,j\in G} p_{ij}^{(m)} \tau_{ij}/N$, which represents the expectation of τ_{ij} over all possible choices of i and j in the stationary distribution of the random walk. According to reference [1], for $m_1, m_2 \geq 0$, we have

$$\left\langle \sum_{i\in G} \frac{1}{N} x_i \cdot (x_i^{(m_1)} - x_i^{(m_2)}) \right\rangle^{\circ} = \frac{\tau^{(m_2)} - \tau^{(m_1)}}{2N}. \tag{S13}$$

Let $\tau_{ii}^{+} = \frac{1}{1-\theta} + \sum_{j\in G} p_{ij}\tau_{ij}$ be the expected remeeting time in the discrete-time random walk, we have

$$\tau^{(m+1)} - \tau^{(m)} = \sum_{i\in G} \frac{1}{N} p_{ii}^{(m)} \tau_{ii}^{+} - \frac{1}{1-\theta}, \tag{S14}$$

where $p_{ii}^{(m)}$ denotes the probability that an m-step random walk terminates at its starting position i. In particular, for regular networks, we have $\tau_{ii}^{+} = N/(1-\theta)$ and $p_{ii}^{(m)} = p^{(m)}$ for all $i \in G$ [1].

Note that for random walks on regular networks, $p^{(1)}$ is the edge weight of self-loops, and $p^{(2)} = 1/\kappa$, where κ is the Simpson degree of the network. Particularly, for unweighted regular network, $p^{(1)} = 0$, and $\kappa = d$ [1, 2]. The parameter $p^{(3)}$ represents the probability that a random walk goes through a triangle loop and terminates at the starting position. The corresponding probability of such a single triangle random walk is $1/d^3$. Thus, the number of triangle loops is $p^{(3)}/(2/d^3)$. Moreover, this quantity is also related to the global clustering coefficient $\mathcal{C}$ [3] since

$$\mathcal{C} = \frac{\text{number of closed triplets}}{\text{number of all triplets}},$$

which is the average of all local clustering coefficients. Based on this, we have

$$\mathcal{C} = \frac{p^{(3)}/(2/d^3)}{d(d-1)/2} = \frac{p^{(3)} d^2}{d-1}.$$

Now, we have that for regular networks with degree d,

$$p^{(1)} = 0, \quad p^{(2)} = \frac{1}{d}, \quad p^{(3)} = \frac{d-1}{d^2}\mathcal{C}. \tag{S15}$$

Substituting Eqs. (S2), (S3), (S13), (S14), and (S15) into condition (S10), we can simplify the condition for

cooperation to be favoured over defection. For donation games, we have

$$\begin{aligned}\left\langle \frac{\partial}{\partial\delta}D\right\rangle^{\circ} =& \frac{1}{2N}\left\{\left(2\theta+(1-\theta)\frac{(s-1)d}{s(d-1)}\right)[b(Np^{(1)}-1)-c(Np^{(0)}-1)]+(1-\theta)\frac{(s-1)d}{s(d-1)}[b(Np^{(2)}-1)-c(Np^{(1)}-1)]\right\}\\ =& \frac{1}{2N}\left\{b\left[N\left((1-\theta)\frac{(s-1)}{s(d-1)}\right)-2(1-\theta)\frac{(s-1)d}{s(d-1)}-2\theta\right]\right.\\ &\left.-c\left[N\left((1-\theta)\frac{(s-1)d}{s(d-1)}+2\theta\right)-2(1-\theta)\frac{(s-1)d}{s(d-1)}-2\theta\right]\right\}. \end{aligned} \tag{S16}$$

Solving $\langle \frac{\partial}{\partial\delta}D\rangle^{\circ} > 0$, we recover Eq. (4) for the success of cooperators under donation games.

Similarly, for public goods games, we have

$$\begin{aligned}\left\langle \frac{\partial}{\partial\delta}D\right\rangle^{\circ} = \frac{(d+1)}{2N}&\left\{r\left[N\frac{(\mathcal{C}(d-1)+2)(1-\theta)\frac{(s-1)d}{s(d-1)}+(d+1)\left((1-\theta)\frac{(s-1)d}{s(d-1)}+2\theta\right)}{(d+1)^2}-2(1-\theta)\frac{(s-1)d}{s(d-1)}-2\theta\right]\right.\\ &\left.-N\left((1-\theta)\frac{(s-1)d}{s(d-1)}+2\theta\right)+2(1-\theta)\frac{(s-1)d}{s(d-1)}+2\theta\right\}. \end{aligned} \tag{S17}$$

The condition of the success of cooperation thus can be calculated as $\rho_{\mathrm{C}}-\rho_{\mathrm{D}} = 2\delta\left\langle \frac{\partial}{\partial\delta}D\right\rangle^{\circ}+\mathcal{O}(\delta^2) > 0$.

For particular regular networks, the clustering coefficients can be calculated explicitly: $\mathcal{C}=0$ (lattice, $d\le 4$), $\mathcal{C}=2/5$ (lattice, $d=6$), $\mathcal{C}=3/7$ (lattice, $d=8$), and $\mathcal{C}=\frac{(d-1)^2}{3Nd}$ (random regular network, $N\to\infty$) [4].

Supplementary Note 4: The threshold of the network clustering coefficient

To present the existence of the threshold of the clustering coefficient on regular networks, we construct a series of regular networks with an increase of the corresponding clustering coefficient.

For a network with a node set $V=\{0,...,N-1\}$, we first construct a ring regular network, where the nodes are sequentially connected end-to-end (see Supplementary Fig. S7). We designate the node preceding node i as $i-1$ and the succeeding node as $i+1$. Consequently, we seek to establish a numerical arrangement relationship such that the node preceding 0 is $N-1$ (i.e., $i-1=N-1$ if $i=0$), and the node succeeding $N-1$ is 0 (i.e. $i+1=0$ if $i=N-1$). Then we add more links and construct a d-regular network, where the set of neighbours for node i is

$$\zeta_i=\{i+\iota_1,i+\iota_2,i+\iota_3,...,i+\iota_d\},$$

where $\iota_1=1,\iota_2=-1$, $-N<\iota_j<N$ and $i+\iota_j\neq i+\iota_k$, for $j\neq k$. Then if for any $j\in\zeta_i$, $\zeta_i\cap\zeta_j=\varnothing$, the clustering coefficient $\mathcal{C}$=0. Following the above procedure, we know that the maximum degree of the network is $d=N/2$, and if the degree is further increased, the clustering coefficient will no longer be zero.

To generate d-regular networks with increasing clustering coefficients, we consider $N=50$, and let $\zeta_i=\{i+1,i-1,i+3,i-3,i+5,i-5,i+7,i-7,i+9,i-9,i+11,i-11\}$. Thus, we have $d=12$. Then, we rewire the edges in an organized manner. From node 1 to node N, we execute the following operations sequentially: remove edge $(i,i+3)$ and $(i+1,i+6)$, and add edge $(i,i+6)$ and $(i+1,i+3)$ (see Supplementary Fig. S7). For every two nodes traversed, we save the structure of the network and assign a corresponding network identifier to generate a series of d-regular networks with increasing clustering coefficients (Supplementary Fig. S8a). In the following, we conduct simulations on the first eleven networks (including the first network with $\mathcal{C}=0$). Using the data points from simulations, we calculate the critical value of r, r^*, by solving the equations resulting from

a linear regression of $\rho_{\mathrm{C}} - \rho_{\mathrm{D}}$ as a function of r (Fig. 3). As shown in Supplementary Fig. S8b, we present that there exists a threshold of the clustering coefficient on regular networks where the impact of social information reverses.

Supplementary figures

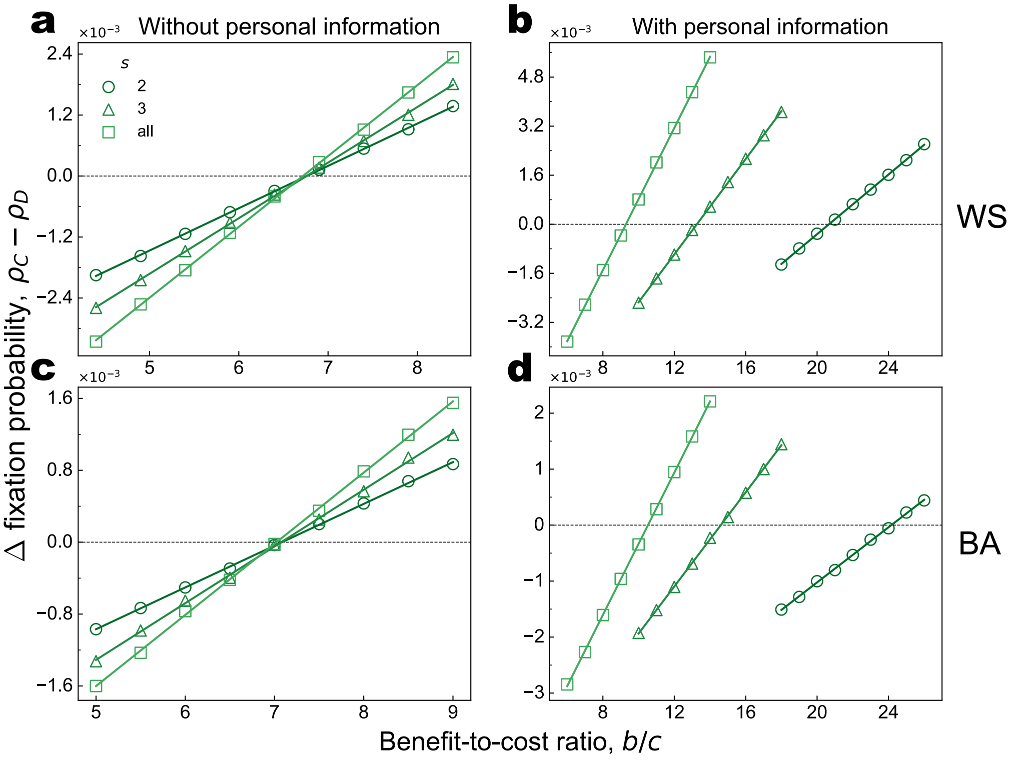

Supplementary Figure S1: Effect of incomplete information on the fixation of cooperation on heterogeneous networks under pairwise social dilemmas. We plot fixation probability difference $\rho_C - \rho_D$ of pairwise social dilemmas as a function of the benefit-cost ratio b/c on Barabási-Albert networks (BA) and small-world networks (WS). Markers are from numerical simulations and lines from the corresponding linear curve fitting. For both networks $N = 100, \langle k \rangle = 6$, and $p = 0.3$ for the small world network. The minimum degrees of both networks are 3, and $1 < s \leq 3$. The simulations when all social information is known are also performed (all). The critical values of $(b/c)^*$ are the same without personal information (**a**, **c**), and different when personal information is considered (**b**, **d**).

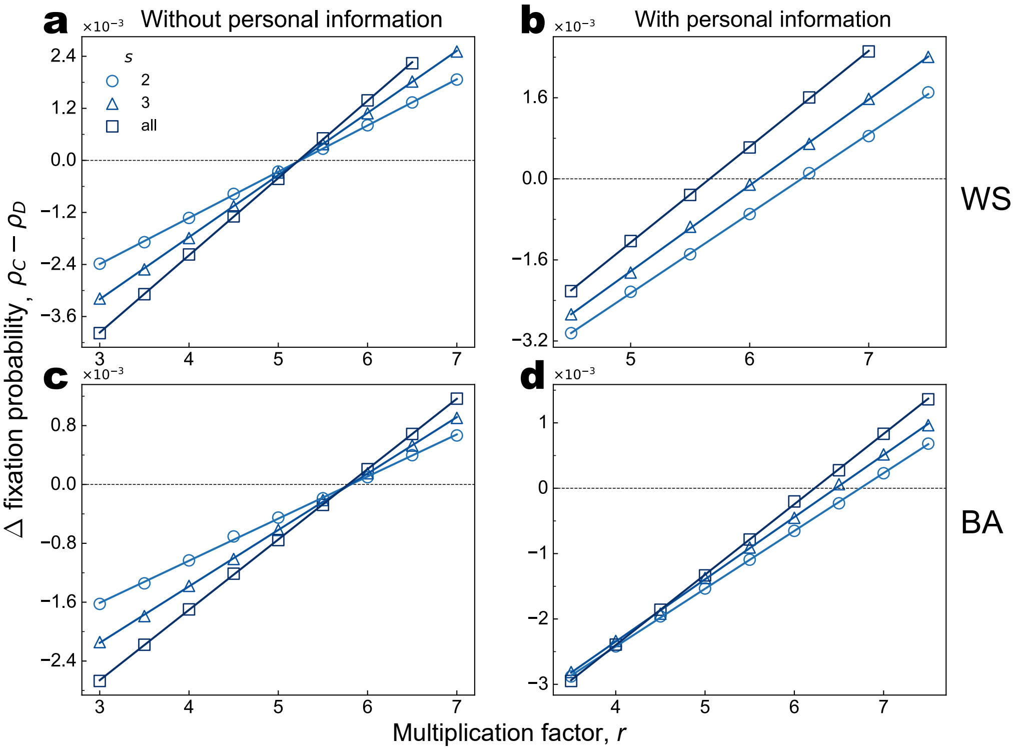

Supplementary Figure S2: Effect of incomplete information on the fixation of cooperation on heterogeneous networks under group social dilemmas. We plot fixation probability difference $\rho_C - \rho_D$ of group social dilemmas as a function of the multiplication factor r on Barabási-Albert networks (BA) and small-world networks (WS). Markers are from numerical simulations and lines from the corresponding linear curve fitting. For both networks $N = 100, \langle k \rangle = 6$, and $p = 0.3$ for the small world network. The minimum degrees of both networks are 3, and $1 < s \leq 3$. The simulations when all social information is known are also performed (all). The critical values of r^* are the same without personal information (**a**, **c**), and different when personal information is considered (**b**, **d**).

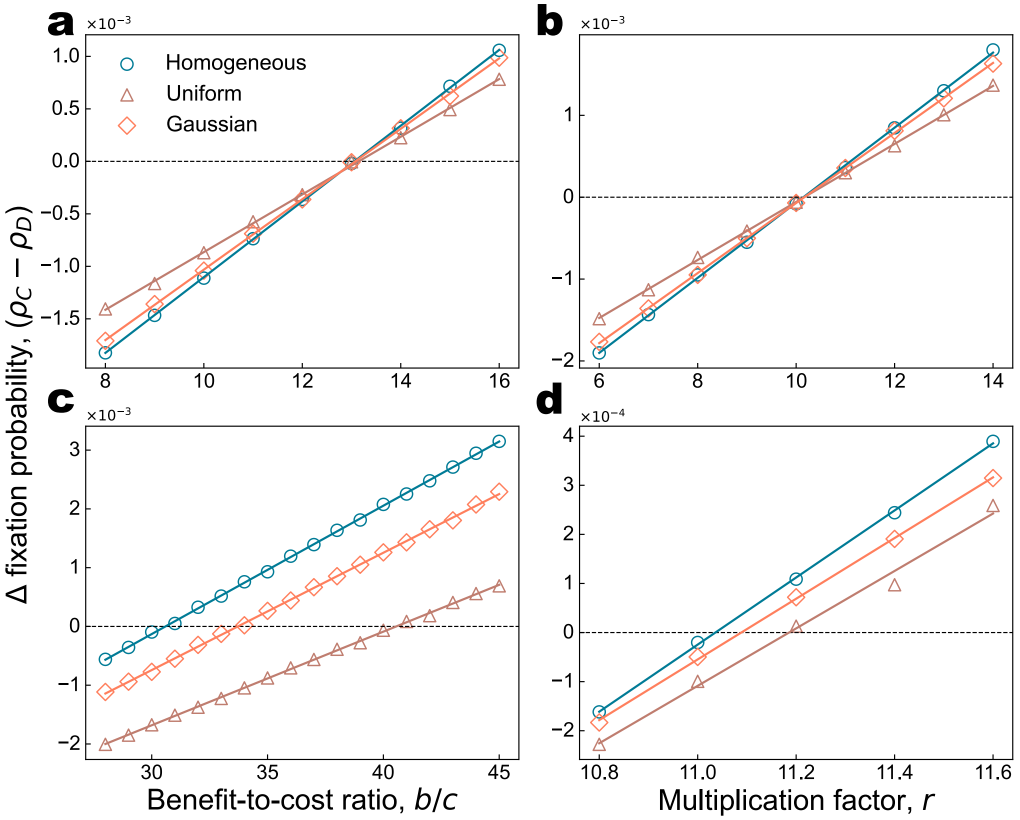

Supplementary Figure S3: The fixation of cooperation on Barabási-Albert networks with heterogeneous social information. For three different distributions of social information (homogeneous, uniform, and Gaussian), we present the fixation probability difference $\rho_C - \rho_D$ for pairwise (**a** and **c**) and group (**b** and **d**) social dilemmas on Barabási-Albert networks [55]. The distributions are the same as those in Fig. 5 of the main text. Here, markers are from numerical simulations and lines are from the corresponding linear curve fitting. When personal information is not considered ($\theta = 0$), we find that information heterogeneity does not change the critical values (i.e. $(b/c)^*$ and r^*) under different distributions for pairwise (**a**) and group (**b**) interactions. When an individual's personal information is taken into account, the results change (**c** and **d**), showing that the homogeneous distribution generates the smallest value of $(b/c)^*$ and r^*. Note that these results are consistent with those in Fig. 5 which focuses on regular networks. Here, the parameters are $N = 100, \langle d \rangle = 10, \delta = 0.01$.

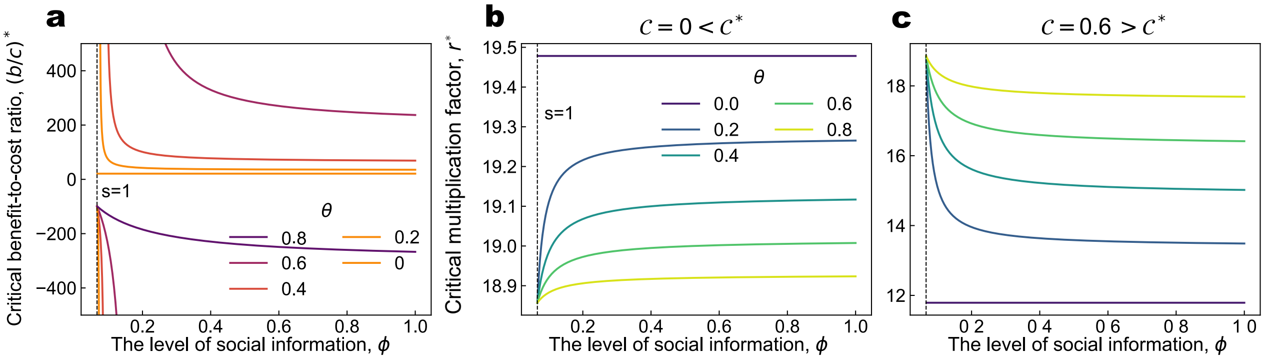

Supplementary Figure S4: Impact on the level of incomplete information on the fixation of cooperation. **a**, The critical $(b/c)^*$ as a function of the level of social information $\phi = s/d$ for donation game of different personal information θ on regular networks with $N = 100, d = 15$. When $\theta = 0$, social information does not influence the critical value. When $\theta \neq 0$, there exists a critical value ϕ_{cr}. The curves diverge at $\phi = 1/d$ and go to negative infinity when approaching ϕ_{cr}, and then decrease from infinity. Larger θ leads to larger $(b/c)^*$. **b**, **c**, The critical r^* as a function of the level of social information ϕ when $\mathcal{C} = 0$ and $\mathcal{C} = 0.6$ on regular networks with $N = 100, d = 30$. When $\theta = 0$, social information does not influence the critical value. Note that for $\theta \neq 0$, all curves diverge at $\phi = 1/d$, i.e., $s = 1$. Larger θ leads to larger r^* when $\mathcal{C} > \mathcal{C}^*$. But this is completely reversed when $\mathcal{C} < \mathcal{C}^*$.

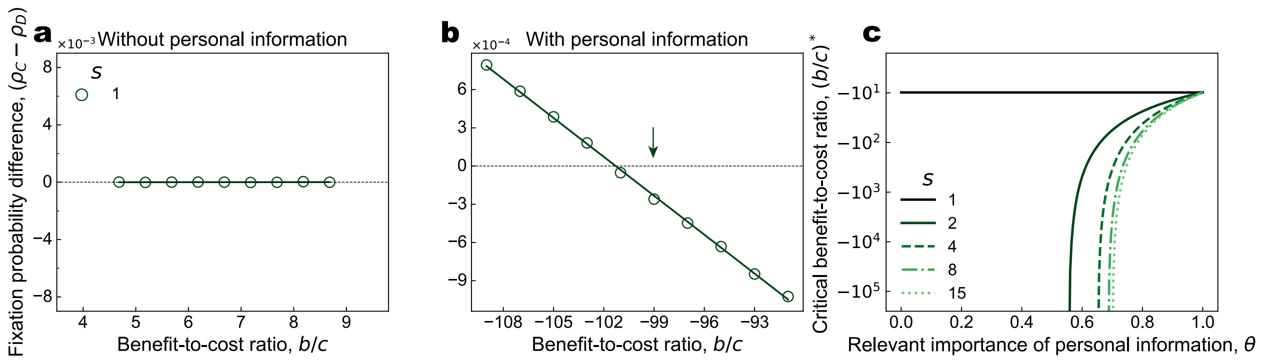

Supplementary Figure S5: Effect of incomplete information on the fixation of cooperation under pairwise social dilemmas when $s = 1$**.** We plot fixation probability difference $\rho_C - \rho_D$ as a function of benefit-cost ratio b/c when $\theta = 0$ (**a**) and $\theta = 1/(s+1)$ (**b**) under $s = 1$ and weak selection ($\delta = 0.01$) when $N = 100$, $d = 6$. **a**, If individuals ignore their personal information ($\theta = 0$), $s = 1$ makes the evolutionary process a neutral drift, resulting in $\rho_{\mathrm{C}} = \rho_{\mathrm{D}}$. **b**, When individuals treat social and their personal information equally important ($\theta = 1/(s+1)$), $s = 1$ makes the update rule reduce to the pairwise-comparison rule, leading to $(b/c)^* = 1 - N$. **c**, The $(b/c)^*$ for the donation game under different social information $s \neq 1$ when θ is larger than the point where $(b/c)^*$ goes to infinity in Fig. 2 of the main text. The curves of $s \neq 1$ converge from negative infinity as $\theta \to 1$. When $s = 1$, $\theta \neq 0$, personal information does not influence the evolutionary process, which echoes panel **b** for $(b/c)^* = 1 - N$. Here, $N = 100, d = 15$.

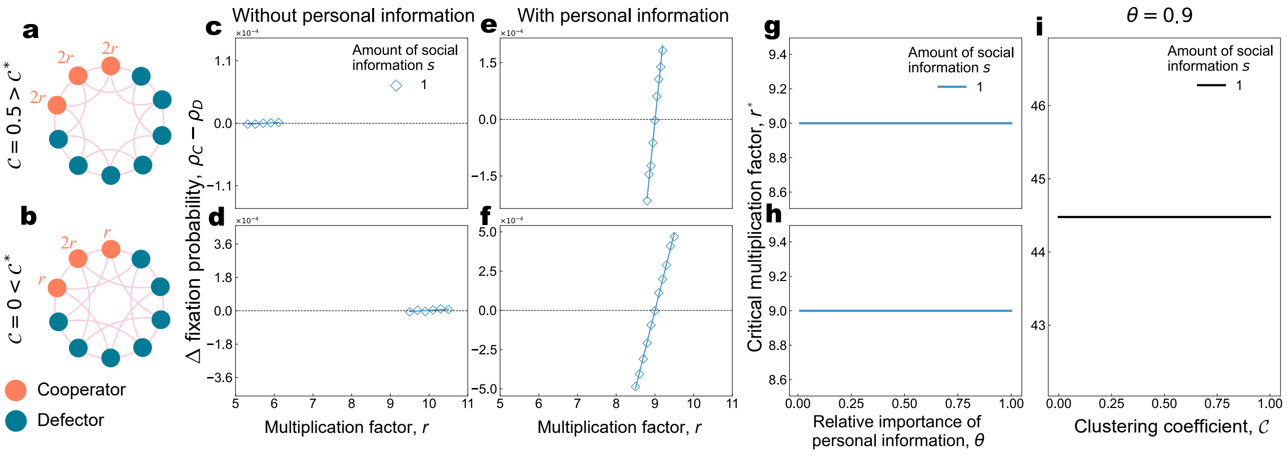

Supplementary Figure S6: Effect of incomplete information on the fixation of cooperation under group social dilemmas when $s = 1$. We consider two different regular networks with different clustering coefficients. **a**, On the network with $\mathcal{C} = 0.5$, the payoffs of the public pools organised by cooperators are $2r$. **b**, On the network with $\mathcal{C} = 0$, the payoffs of the group organised by the cooperators on both sides decrease. We plot fixation probability difference $\rho_C - \rho_D$ as a function of multiplication factor r. Here markers are from numerical simulations and lines are from the corresponding linear curve fitting. When individuals ignore their own information ($\theta = 0$), the fixation probabilities of a cooperator and defector are equal ($\rho_{\mathrm{C}} = \rho_{\mathrm{D}}$) (**c, d**). When individuals treat both kinds of information equally ($\theta = 1/(s+1)$), the r^* is the same for different $\mathcal{C}$ (**e, f**). We draw the critical r^* as a function of the weight of personal information θ for the networks in **a**, and **c**. r^* is irrelevant to the clustering coefficient $\mathcal{C}$ and the weight of personal information-information θ (**g, h, i**). For all panels, $c = 1$, other parameters are the same as those in Fig. S5.

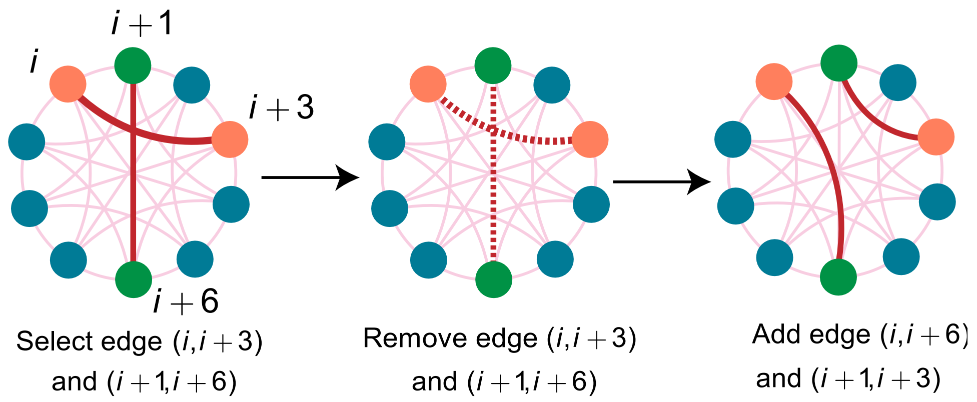

Supplementary Figure S7: Illustration of the construction of regular networks with increasing clustering coefficients. We begin with a regular network that has a clustering coefficient $\mathcal{C} = 0$. In this example, we have $N = 10$, and the set of neighbours of i is $\zeta_i = \{i+1, i-1, i+3, i-3, i+5\}$ (see Supplementary Note 4 for a general description). We traverse each node i and execute the following operations sequentially: remove edge $(i, i+3)$ and $(i+1, i+6)$ and add edge $(i, i+6)$ and $(i+1, i+3)$. As illustrated in this figure, after operations for one node, the clustering coefficient increases from $\mathcal{C} = 0$ to $\mathcal{C} = 0.18$.

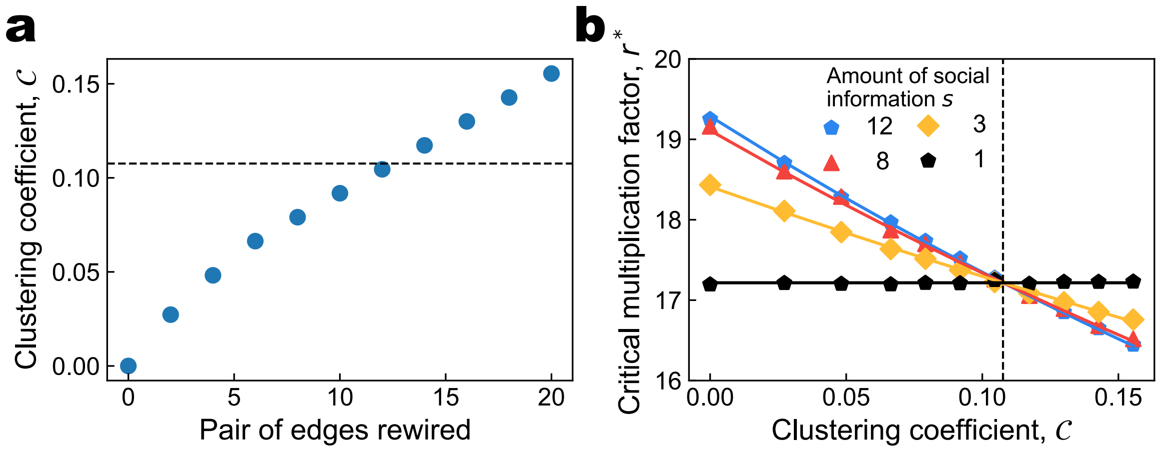

Supplementary Figure S8: The threshold of the clustering coefficient on regular networks. a, The clustering coefficient $\mathcal{C}$ increases with the pair of edges rewired. The dashed line represents the threshold of $\mathcal{C}$, i.e., $\mathcal{C}^*$, as shown in Eq. (7) in the main text. **b**, Taking the situation where individuals treat both kinds of information equally ($\theta = 1/(s+1)$) as an example, we conduct simulations on regular networks with different clustering coefficients $\mathcal{C}$ and find the corresponding r^* by solving the equations resulting from a linear regression of $\rho_{\mathrm{C}} - \rho_{\mathrm{D}}$ as a function of r. We draw r^* as a function of the clustering coefficient $\mathcal{C}$. The data points represent the values of r^* obtained from the linear regression of the simulation results, and the solid lines correspond to the theoretical values of r^* calculated from Eq. (6). The vertical dashed line represents the threshold of $\mathcal{C}$, i.e., $\mathcal{C}^*$, as shown in Eq. (7) in the main text.

Supplementary References

[1] Allen, B., Lippner, G., Chen, Y.-T., Fotouhi, B., Momeni, N., Yau, S.-T. & Nowak, M. A. Evolutionary dynamics on any population structure. *Nature* **544**, 227–230 (2017).

[2] Allen, B. & Nowak, M. A. Games on graphs. *EMS Surv. Math. Sci.* **1**, 113–151 (2014).

[3] Watts, D. J. & Strogatz, S. H. Collective dynamics of ‘small-world’networks. *Nature* **393**, 440–442 (1998).

[4] Su, Q., Li, A., Wang, L. & Eugene Stanley, H. Spatial reciprocity in the evolution of cooperation. *Proc. R. Soc. B* **286**, 20190041 (2019).
